# Clinical outcomes of reverse sural artery flap with the addition of lateral sural nerve and fasciocutaneous pedicle: Double sural nerve branches technique

**DOI:** 10.1016/j.jpra.2026.08.027

**Published:** 2026-08-25

**Authors:** Ryu Igaki, Tomohiro Yasuda, Shinsuke Takagi, Yuto Murakami, Yuki Samejima, Keikichi Kawasaki, Koichi Kadomatsu

**Affiliations:** a Department of Plastic and Reconstructive Surgery, Showa Medical University School of Medicine, Japan; b Department of Orthopedic Surgery, Showa Medical University Fujigaoka Hospital, Japan; c Department of Orthopedic Surgery, Teikyo University Hospital, Mizonokuchi, Japan; d Department of Orthopedic Surgery, Yokohama Shintoshi Neurosurgical Hospital, Japan; e Department of Orthopedic Surgery, Showa Medical University Northern Yokohama Hospital, Japan; f Department of Plastic and Reconstructive Surgery, Showa Medical University Fujigaoka Hospital, Japan

**Keywords:** Reverse sural artery flap, Fasciocutaneous pedicle, Lateral sural nerve

## Abstract

**Background:**

Soft tissue reconstruction of the distal lower extremity and foot is challenging for reconstructive surgeons, and reconstruction options are limited. Although reverse sural artery flap (RSAF) is often used in such cases, the incidence of complications requiring treatment is 14%. To reduce the incidence of complications, we performed the double sural nerve branches (DSNB) technique, a modified RSAF incorporating the medial and lateral sural nerves and a fasciocutaneous pedicle.

**Methods:**

This was a retrospective case series conducted at a single institution. In total, 22 patients who underwent reconstruction using the DSNB technique for soft tissue defects in the distal leg and foot, including traumatic and postoperative cases, between April 2013 and April 2023 were included.

**Results:**

The flap survived in all 22 patients, with no cases of partial or total flap loss. Marginal necrosis occurred in two patients (9.1%), both of whom also experienced venous congestion; however, neither condition required additional surgical intervention.

**Conclusion:**

The complication rate associated with the DSNB technique was 9.1%, with a 100% flap survival rate. The DSNB technique may represent a useful modification of the RSAF; however, further studies are warranted to clarify its clinical utility.

## Introduction

Soft tissue defects in the distal lower leg and foot caused by trauma often require reconstruction to cover exposed bone and tendons. The reverse sural artery flap (RSAF) is a commonly used method for reconstruction in this region. RSAF is a distally based fasciocutaneous or adipofascial flap that includes the medial sural nerve, the medial superficial sural artery, and the small saphenous vein. The medial superficial sural artery communicates distally with the perforating branch of the peroneal artery.

The procedure is widely used because it is relatively simple and does not require microsurgery. Although the survival rate of RSAF is 95.2%, the incidence of complications requiring treatment is reported to be 14%. Of the complications, 75.3% were venous congestion, 63% were epidermal loss, and 55.9% were margin necrosis.^1^

To reduce the incidence of complications, we performed the double sural nerve branches (DSNB) technique, a modified RSAF incorporating the medial and lateral sural nerves together with a fasciocutaneous pedicle.

In this study, we evaluated the clinical outcomes of the DSNB technique and explored potential predictors of postoperative complications.

## Patients and methods

This study was conducted in accordance with the ethical principles outlined in the Declaration of Helsinki and its subsequent amendments. The research protocol was reviewed and approved by the Ethics Committee of Showa Medical University (Approval No. 2024-228-A).

This was a retrospective case series conducted at a single institution. The manuscript was checked against the Strengthening the Reporting of Observational Studies in Epidemiology (STROBE) checklist, which is provided the Supplementary Appendix. Patients who underwent reconstruction using the DSNB technique for soft tissue defects in the distal leg and foot, including traumatic and postoperative cases, were retrospectively identified between April 2013 and April 2023. A total of 22 patients were included. Patients who were followed up for <1 year were excluded. Free flaps are also indicated for soft tissue defects in the distal lower leg to the foot due to trauma. The decision to use the DSNB technique was based on predefined clinical criteria at our institution: (1) a defect measuring <15 cm in length and 7 cm in width, with the distal limit of coverage extending to the metatarsal region and (2) adequate blood flow in the donor area confirmed by ultrasonography. Within this anatomically eligible group, the DSNB technique was preferentially selected for patients in whom free flap reconstruction was considered less suitable because of advanced age, dementia, severe arteriosclerotic disease, or calcification of the recipient vessels. The evaluation items in this study included medical history (e.g., peripheral artery disease, diabetes mellitus), smoking history, age, etiology, defect size, defect site, venous congestion, flap necrosis, and flap infection. All clinical data were retrospectively obtained from medical records; defect size was assessed based on operative findings. Medical records were also reviewed for donor-site morbidity, including documented donor-site pain, clinical symptoms suggestive of symptomatic neuroma, and any additional treatment related to donor-site sensory complaints. The presence of venous congestion in the flap was evaluated on the first postoperative day. Flap necrosis was categorized as marginal necrosis, partial flap loss, or total flap loss according to previously reported criteria. Marginal necrosis was defined as limited tip or edge necrosis managed conservatively, whereas partial flap loss was defined as tissue necrosis requiring additional surgical intervention.^2^ The primary outcomes were flap necrosis and venous congestion. Potential predictors included medical history, smoking history, and the presence of a fibular fracture. Associations between these factors and flap necrosis were evaluated.

### Surgical technique

**Fig. 1:**
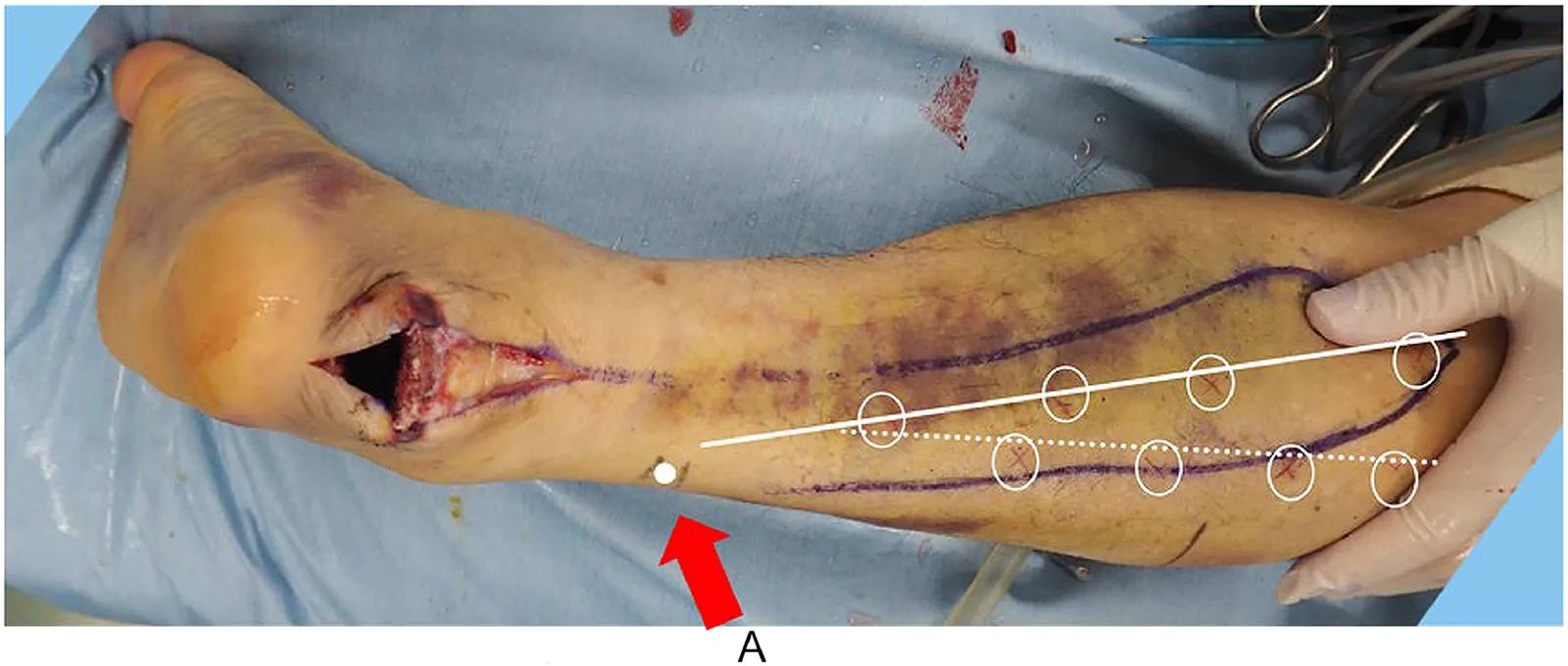
Flap design and marking with ultrasonography. The peroneal perforator was identified via ultrasonography and marked as Point A. The medial sural nerve and median superficial sural artery were marked (white circles on solid line a). The flap axis was drawn by connecting them to Point A (solid line a). The lateral sural nerve was also marked (dashed line b).

The surgery was performed with all patients in the prone position. The medial superficial sural artery could be visually confirmed without the use of tourniquet. The flap design was preoperatively designed using ultrasonography (sound Doppler and color Doppler). The peroneal perforator was identified by ultrasonography between the Achilles tendon and the fibula and marked as Point A for the pivot point (Fig. 1: red arrow). The peroneal perforator was identified 5–7 cm from the tip of the lateral malleolus. The medial sural nerve and its accompanying medial superficial sural artery were identified by ultrasonography and marked as multiple points (Fig. 1: white circles on solid line a). The flap axis (Fig. 1: solid line a) was drawn by connecting these points to Point A (the pivot point).

Next, the lateral sural nerve was confirmed by ultrasonography and marked (Fig. 1: dotted line b). At the base of the flap, a fasciocutaneous pedicle with a width of at least 3 cm was secured at the pivot point. It was designed to create a fasciocutaneous pedicle rather than an adipofascial pedicle. The thoroughly debrided soft tissue defect area was traced onto clean paper, and the island flap was designed accordingly. The flap dimensions were intentionally enlarged by 2–3 cm in length and 1–1.5 cm in width relative to the size of the defect.

**Fig. 2:**
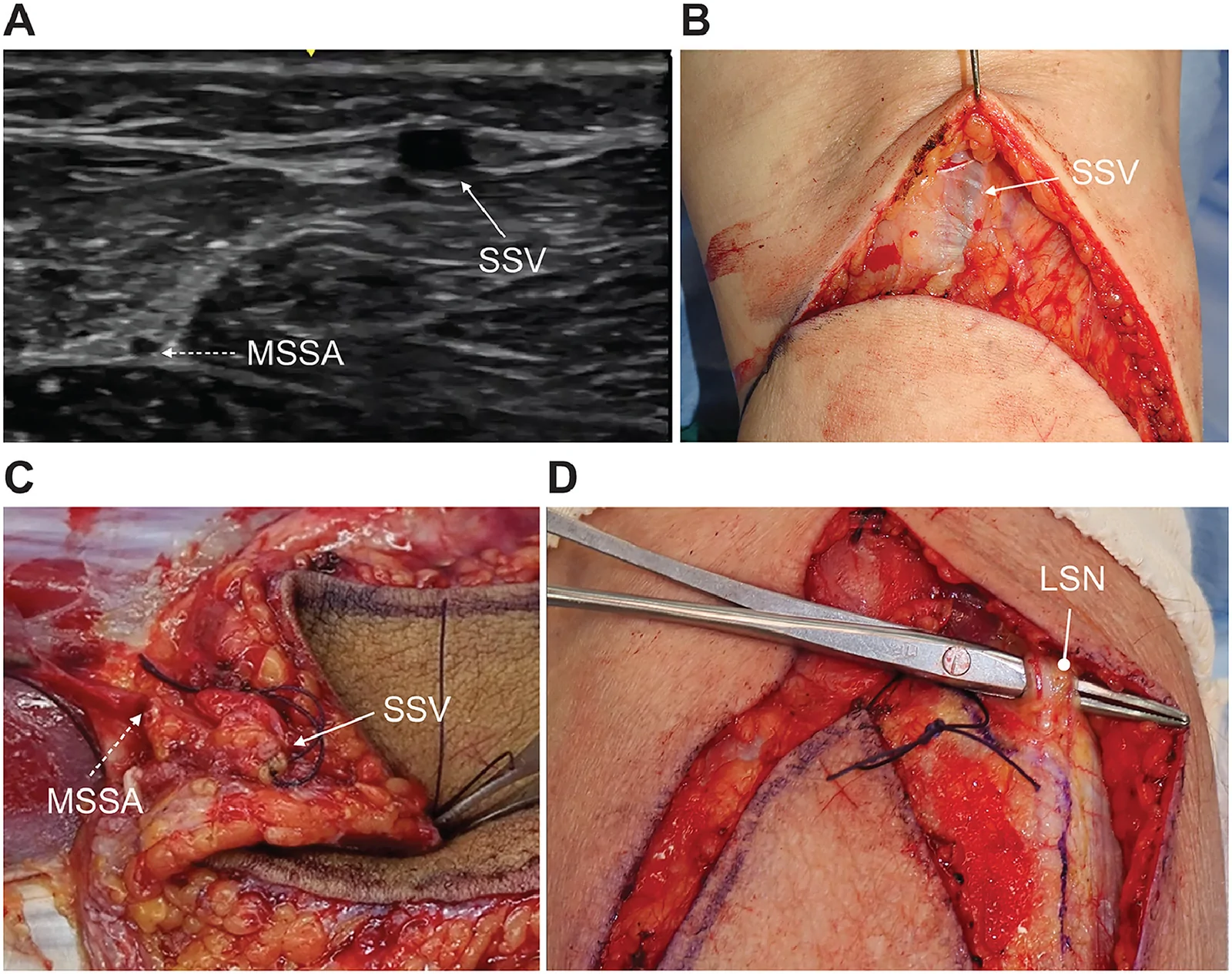
(a) Ultrasonography image of the proximal side of the flap. The small saphenous vein (solid arrow) was surrounded by the deep fascia and underlying fascial sheath. The medial sural nerve and medial superficial sural artery ran between the medial and lateral heads of the gastrocnemius muscle (dashed arrow). (b) During the surgical procedure, it was observed that the small saphenous vein traversed beneath the deep fascia (solid arrow). (c) The medial sural nerve (dashed arrow) and the medial superficial sural artery (dashed arrow) were dissected, transected, and included in the flap, which was then elevated. The medial superficial sural artery (dashed arrow) was visible under surgical loupe. (d) The lateral sural nerve running beneath the fascia (solid line) was included in the flap. SSV, small saphenous vein; MSSA, medial superficial sural artery; MSN, medial sural nerve; LSN, lateral sural nerve.

Flap elevation was started by making an incision proximal to the designed flap. In the proximal one-quarter of the lower leg, the small saphenous vein runs under the deep fascia, and the fascial sheath is present in the deep layer.^3^ The medial superficial sural artery and medial sural nerve run between the medial and lateral heads of the gastrocnemius muscle. The positional relationship of the small saphenous vein, the medial superficial sural artery, and the medial sural nerve was confirmed preoperatively by ultrasonography (Fig. 2-a). The small saphenous vein running under the deep fascia was sectioned and included in the flap (Fig. 2-b). After deep dissection between the muscles, the medial superficial sural artery and the medial sural nerve were carefully dissected, transected, and included in the flap. The median superficial sural artery was visible with a surgical loupe (Fig. 2-c). The fascia was elevated, extending approximately 3 cm beyond the skin incision. The lateral sural nerve under the fascia in the proximal part of the lower leg was identified and included in the flap (Fig. 2-d). After transection of the lateral sural nerve, bleeding was observed from its accompanying artery. The medial sural nerve, lateral sural nerve, and medial superficial sural artery were included in the flap, which was elevated in a subfascial plane from the proximal to the distal side. The flap was rotated around the pivot point and sutured to the defect.

If the donor area could not be sutured, a split-thickness skin graft from the thigh was performed. Postoperatively, the flap was bandaged to prevent pressure on the skin flap; however, splint fixation was not performed in all patients. Patients were placed on bed rest for 3 days after surgery.

### Statistical analysis

Statistical analyses were performed using JMP Pro 17 (SAS Institute Inc., Cary, NC, USA). The relationship between complications, smoking history, and fractures with flap necrosis was examined. Because this involved categorical variables, the two-tailed Fisher’s exact test was used for the analysis. Exact 95% confidence intervals (Clopper–Pearson method) were calculated for the major proportions, including flap failure, marginal necrosis, and venous congestion. A p-value of <0.05 was considered statistically significant.

## Results

All 22 patients who underwent soft tissue defect reconstruction using the DSNB technique were followed up for at least 1 year. The mean age of the patients was 51.7 (range: 19–90) years, and 17 were male and five were female. Medical history included peripheral artery disease (PAD) in five patients and type 2 diabetes mellitus (DM) in two. Regarding social history, 10 patients had a history of smoking. Additionally, 10 patients presented with an associated fibular fracture.

**Fig. 3:**
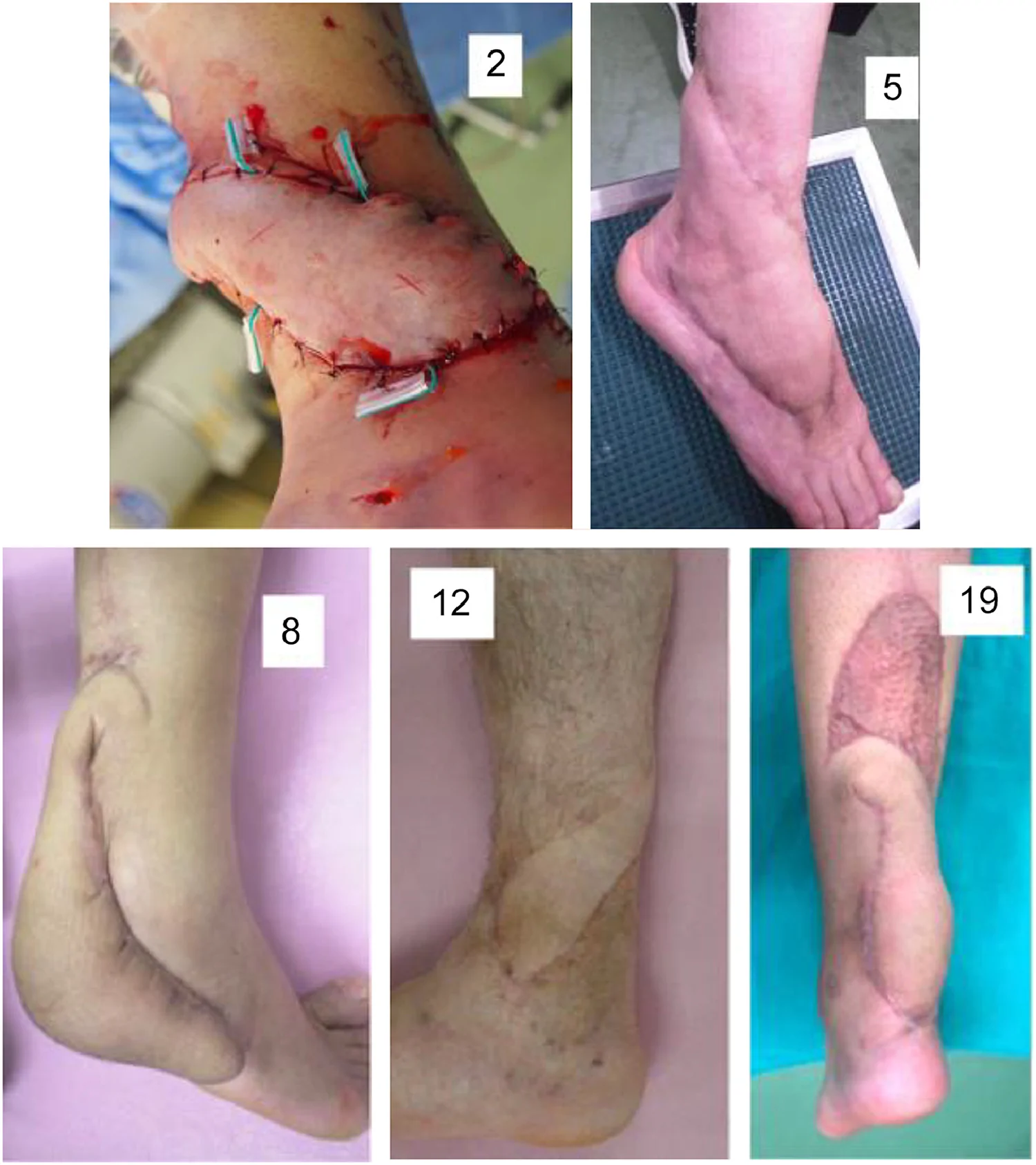
List of the representative cases. The values indicate the case numbers. Venous congestion was observed in case 2. A split-thickness skin graft was performed at the donor site in case 19.

The underlying conditions included open distal tibia fracture in seven patients, open calcaneus fracture in four patients, pilon fracture in three patients, open ankle joint dislocation fracture in two patients, open foot fracture in two patients, soft tissue loss due to wound infection after Achilles tendon rupture surgery in three patients, and soft tissue loss due to wound infection after distal tibia fracture surgery in one patient. In one patient (Case 20), the DSNB technique was selected because free flap reconstruction was considered less suitable owing to advanced age and overall physiological risk. The smallest skin flap measured 4 cm × 4 cm, whereas the largest measured 16 cm × 8 cm. The flap survived in 22 patients, with no cases of partial or total flap loss (0/22, 0.0%; 95% CI, 0.0%–15.4%). Marginal necrosis occurred in two patients (9.1%; 95% CI, 1.1%–29.2%) and did not require additional surgical intervention. The same two patients (9.1%; 95% CI, 1.1%–29.2%) also developed flap congestion, which also did not require treatment. No postoperative infections were observed. The list of cases is presented in Table 1, and representative case images are shown in Fig. 3. A retrospective review of the medical records revealed no documented donor-site pain requiring additional treatment and no symptoms suggestive of symptomatic neuroma. Soft tissue reconstruction was required for defects of the distal lower leg in five patients, of the ankle in eight patients, of the heel in seven patients, and of the dorsum of foot in two patients.

**Table 1:** Profile of the 22 patients.

Patient	Age	Etiology	Flap dimensions (cm × cm)						
				Venous congestion	Necrosis	PAD	DM	Smoking	Fibular fracture
1	48	Pilon fracture	11 × 5		None				+
2	51	Pilon fracture	6 × 5	Slight	Marginal				+
3	64	Pilon fracture	11 × 5	Slight	Marginal		+		+
4	50	Distal tibial open fracture	14 × 5		None			+	+
5	49	Open fracture of the foot	14 × 10		None				
6	31	Open fracture of the foot	14 × 10		None				
7	62	Open calcaneus fracture	5 × 4		None	+	+	+	
8	69	Open calcaneus fracture	16 × 8		None			+	
9	19	Distal tibia open fracture	5 × 4		None				
10	45	Distal tibia open fracture	8 × 5		None			+	+
11	61	Open calcaneus fracture	11 × 5		None			+	
12	53	Distal tibia open fracture	8 × 5		None				+
13	41	Distal tibia open fracture	8 × 6		None				+
14	62	Infection after Achilles tendon repair	6 × 4		None	+		+	
15	42	Open dislocation of the ankle	7 × 5		None	+			
16	56	Open dislocation of the ankle	8 × 5		None			+	+
17	27	Distal tibia open fracture	12 × 8		None			+	+
18	55	Distal tibia open fracture	4 × 4		None			+	
19	24	Infection after Achilles tendon repair	7 × 5		None				
20	90	Postoperative infection after distal tibia fracture	12 × 7		None	+			+
21	62	Infection after Achilles tendon repair	8 × 5		None				
22	77	Open calcaneus fracture	4 × 4		None	+

The incidence of marginal necrosis also varied according to potential predictors. Marginal necrosis occurred in 0 of 5 patients with PAD (0%; 95% CI, 0%–52.2%), 1 of 2 patients with type 2 DM (50%; 95% CI, 1.3%–98.7%; p = 0.1775), and 0 of 10 patients with a smoking habit (0%; 95% CI, 0%–30.8%), and 2 of 10 patients with fibular fractures (20%; 95% CI, 2.5%–55.6%; p = 0.1948). None of these associations reached statistical significance (Table 2).

**Table 2:** Analysis of factors influencing flap necrosis.

Examination of factors affecting skin flap necrosis		
Complications (22 cases)	Marginal necrosis	P-value#
Type 2 diabetes mellitus (2/22)	1 (50%; 95% CI, 1.3%–98.7%)	0.1775
PAD (5/22)	0 (0%; 95% CI, 0%–52.2%)	-
Smoking (10/22)	0 (0%; 95% CI, 0%–30.8%)	-
Fibular fracture (10/22)	2 (20%; 95% CI, 2.5%–55.6%)	0.1948

# Fisher’s exact test. PAD: Peripheral artery disease.

## Discussion

RSAF has been reported to be a safe technique to reconstruct soft tissue defects in the distal lower leg to the foot, particularly in the foot and ankle.^4^ However, the safety of the procedure has also been debated.^2^ Venous congestion, epidermal loss, and tip necrosis of the flap are common complications of RSAF.^1^ To minimize complications, we developed the DSNB technique, a modified RSAF incorporating the medial and lateral sural nerves and a fasciocutaneous pedicle. All 22 patients who underwent reconstruction using the DSNB technique were retrospectively examined.

No cases of partial or total flap loss were observed. Marginal necrosis occurred in two patients (9.1%), both of whom also experienced venous congestion. No statistically significant associations were observed between DM, fibular fracture, and postoperative complications. Additionally, no complications occurred among patients with PAD or a history of smoking. No infection was observed. A systematic review including 218 articles and a total of 5,145 RSAF cases reported outcomes for both classic unmodified and modified techniques. In the classic fasciocutaneous unmodified RSAF, the total flap failure rate was 5.1%, accompanied by a relevant complication rate of 14.8%. Conversely, modified techniques—such as delayed two-stage operations, combination with a gastrocnemius muscle cuff, supercharged venous drainage, and skin-sparing adipofascial flaps with mesh grafts—achieved a lower total failure rate of 3.6% and a complication rate of 9.1%.^1^ In the present series, the complication rate was 9.1%, with no cases of total flap failure. The detailed dissection of the RSAF revealed that it is nourished by the superficial sural artery, which runs alongside the sural nerve. It was reported as a distally based neuroskin flap of sural nerve by Masquelet et al. in 1992. They also described that the superficial sural artery communicates distally with a perforating branch of the peroneal artery.^5^ Because of this anatomical relationship, the RSAF may be at increased risk of necrosis and other flap-related complications in the presence of a fibular fracture. However, the present study did not demonstrate a significant association between fibular fracture and postoperative flap-related complications. Although preoperative ultrasonography may facilitate identification of the peroneal perforators, its clinical utility was not evaluated in the present study. In addition, given the limited sample size, larger studies are required to clarify the effect of fibular fracture on postoperative flap-related complications. In the present study, we performed a DSNB technique including the medial and lateral sural nerves within the flap. The cutaneous nerve possesses two vascular network systems: an intrinsic neurocutaneous vascular system and an extrinsic neurocutaneous vascular system. The extrinsic neurocutaneous system gives off branches not only to the nerve but also to the skin. All cutaneous nerves have an intrinsic neurocutaneous vascular system along their entire length, but they do not always have an extrinsic neurocutaneous vascular system. It has been reported that the sural nerve and lateral sural cutaneous nerve possess a continuous extrinsic neurocutaneous vascular system along their entire course.^6^ Similar vascular structures have also been reported in the lateral femoral cutaneous nerve and the anterior cutaneous branches of the femoral nerve, and these systems have been shown to have numerous anastomoses with subcutaneous vessels.^7^ It has also been reported in other studies that the accompanying artery of the lateral sural nerve gives off branches to the skin.^3^ Adipofascial flaps based solely on the lateral sural nerve as a single sural nerve branch have also been reported.^8^ Based on these reports, including both the medial and lateral sural nerves within the flap is expected to enhance its vascularity. Intraoperatively, favorable retrograde blood flow was observed after incision of the lateral sural nerve. Additionally, several anatomical variations in the course of the lateral sural nerve have been reported, including types in which only the medial sural nerve is present.^9^ In the present study, the lateral sural nerve was included within the flap in all cases. A potential drawback of the DSNB technique is increased donor-site sensory morbidity because both the medial and lateral sural nerves are included in the flap. In the present retrospective study, donor-site morbidity was not formally evaluated. However, a retrospective review of the medical records revealed no cases requiring additional treatment for donor-site pain and no documented symptoms suggestive of painful neuroma. Donor-site sensory disturbance has also been reported after conventional sural nerve harvest. Long-term follow-up studies have shown that although sensory disturbance may persist, its impact on activities of daily living and work is generally mild, and patient satisfaction remains high.^10^ Therefore, donor-site morbidity associated with the DSNB technique may not differ substantially from that associated with conventional sural nerve harvest. However, because donor-site sensory outcomes were not quantitatively assessed in the present study, this hypothesis remains speculative and requires confirmation in prospective studies. We designed the flap with a 3 cm fasciocutaneous pedicle to facilitate venous drainage. Additionally, the adipofascial tissue was included in the flap by extending beyond the skin incision. Sugg et al. reported that RSAFs designed as skin islands with a narrow fascial pedicle experienced a 42% rate of congestion requiring leech therapy, whereas no such congestion was observed in two additional designs aimed at improving venous drainage: RSAFs raised as adipofascial flaps with a 4‐cm pedicle and RSAFs designed with an additional 4‐cm skin pedicle.^11^ In studies using rats, it has also been reported that flaps with a skin pedicle are beneficial for venous drainage.^12^

The maximum flap size in the present series was 16 cm in length and 8 cm in width. The indication for the DSNB technique was predefined as defects measuring <15 cm in length and 7 cm in width, with the distal limit of coverage extending to the metatarsal region. The flap was intentionally designed to be 2–3 cm longer and 1–1.5 cm wider than the defect; therefore, flap dimensions could exceed the predefined defect-size criteria.

Large skin flaps were designed proximal to the popliteal fossa, similar to the extended RSAF reported by Ramesha et al.^13^ However, because such large flaps require skin grafts at the donor site, free flaps are also considered. As depicted in Fig. 3, the appearance after split-thickness skin grafting is often affected by hyperpigmentation, which may compromise cosmetic outcomes. Therefore, when a flap size requiring donor-site skin graft rather than primary closure is anticipated, the indication for the DSNB technique should be carefully considered, particularly in women and children, and free flap reconstruction should also be considered. Appropriate size-based indications require further study. The present study has some limitations. First, this was not a comparative study between conventional RSAF and the DSNB technique, precluding conclusions regarding their relative effectiveness. Second, the sample size was small, and no a priori power analysis or sample size calculation was performed. Therefore, the study may have been underpowered to detect clinically meaningful differences in complication rates, and the absence of statistically significant associations should not be interpreted as evidence of no effect. Third, the single-institution retrospective design and institution-specific indications for the DSNB technique may have introduced selection bias and limited the generalizability of the findings. The cohort included patients with different physiological risk profiles, but the limited sample size precluded meaningful stratified analysis; therefore, outcomes across different risk groups could not be evaluated. Moreover, the cases of venous congestion occurred early in the series, suggesting that a learning curve and other surgeon-related factors may have influenced the outcomes. Therefore, the favorable outcomes cannot be attributed solely to the DSNB technique. Finally, donor-site sensory morbidity was not formally or quantitatively assessed. Although no donor-site pain requiring treatment or symptoms suggestive of painful neuroma were documented, subtle sensory disturbances and patient-reported morbidity may have been underrecognized.

## Conclusion

Soft tissue reconstruction from the distal lower leg to the heel and dorsal foot was performed using the DSNB technique, a modified RSAF incorporating the medial and lateral sural nerves and a fasciocutaneous pedicle.

In this series, no cases of partial or total flap loss were observed, and marginal necrosis occurred in 9.1% of patients without requiring additional surgical intervention. The DSNB technique may represent a useful modification of the RSAF without requiring additional technically demanding procedures. However, further studies are required to clarify its clinical utility.

## Declaration of generative AI in scientific writing

ChatGPT (OpenAI, San Francisco, CA, USA) was used solely to assist with English-language editing and translation. The authors reviewed and edited the content as necessary and take full responsibility for the final manuscript.

## Funding

This research did not receive any specific grant from funding agencies in the public, commercial, or not-for-profit sectors.

## Data availability

The datasets used and/or analyzed during the current study are available from the corresponding author on reasonable request.

## Declaration of competing interest

None.

## Appendix Supplementary materials

Image, application 1
