# Supplementary material for: Clinical outcomes of reverse sural artery flap with the addition of lateral sural nerve and fasciocutaneous pedicle: Double sural nerve branches technique
Source: JPRAS Open. 2026 Aug 25;52:275–81. doi: 10.1016/j.jpra.2026.08.027 (PMC13579141; doi:10.1016/j.jpra.2026.08.027)
Supplement: Supplementary file 1 [file mmc1.docx]

STROBE Statement—checklist of items that should be included in reports of observational studies

|  | Item No. | Recommendation | Page  No. | Relevant text from manuscript |
| --- | --- | --- | --- | --- |
| **Title and abstract** | 1 | (*a*) Indicate the study’s design with a commonly used term in the title or the abstract | Page 3 | This was a retrospective case series conducted at a single institution. |
|  |  | (*b*) Provide in the abstract an informative and balanced summary of what was done and what was found | Page 3 | We performed the double sural nerve branches (DSNB) technique. The flap survived in all patients, with a complication rate of 9.1%. |
| Introduction | | | |  |
| Background/rationale | 2 | Explain the scientific background and rationale for the investigation being reported | Page 3 | The reverse sural artery flap (RSAF) is frequently used, the incidence of complications requiring treatment has been reported to be 14%. To reduce the incidence of complications, we performed the double sural nerve branches (DSNB) technique. |
| Objectives | 3 | State specific objectives, including any prespecified hypotheses | Page 3 | In this study, we report the clinical outcomes of the DSNB technique and its relation to fibular fractures and medical history. |
| Methods | | | |  |
| Study design | 4 | Present key elements of study design early in the paper | Page 5 | This was a retrospective case series. |
| Setting | 5 | Describe the setting, locations, and relevant dates, including periods of recruitment, exposure, follow-up, and data collection | Page 5 | This was a retrospective case series conducted at a single institution, including 22 patients who underwent reconstruction using DSNB technique for soft tissue defects in the distal leg and due to trauma between April 2013 and April 2023. |
| Participants | 6 | (*a*) *Cohort study*—Give the eligibility criteria, and the sources and methods of selection of participants. Describe methods of follow-up  *Case-control study*—Give the eligibility criteria, and the sources and methods of case ascertainment and control selection. Give the rationale for the choice of cases and controls  *Cross-sectional study*—Give the eligibility criteria, and the sources and methods of selection of participants | Page 5 | Patients who underwent reconstruction using the DSNB technique for soft tissue defects in the distal leg and foot, including traumatic and postoperative cases, were retrospectively identified between April 2013 and April 2023. |
|  |  | (*b*) *Cohort study*—For matched studies, give matching criteria and number of exposed and unexposed  *Case-control study*—For matched studies, give matching criteria and the number of controls per case |  | Not applicable |
| Variables | 7 | Clearly define all outcomes, exposures, predictors, potential confounders, and effect modifiers. Give diagnostic criteria, if applicable | Page 5 | The evaluation items in this study included medical history (e.g., peripheral artery disease and diabetes mellitus), smoking history, age, etiology, defect size, defect site, venous congestion, necrosis, and flap infection. The primary outcomes were flap necrosis and venous congestion. The presence of venous congestion in the flap was evaluated on the first postoperative day. Flap necrosis was defined as partial necrosis >5 mm from the margin and margin necrosis when <5 mm from the margin. In addition, the requirement for additional procedures, such as debridement and skin grafting, was also assessed. Potential predictors and confounders included medical history, smoking history, and the presence of a fibular fracture. The association between these factors and flap necrosis was examined. |
| Data sources/ measurement | 8* | For each variable of interest, give sources of data and details of methods of assessment (measurement). Describe comparability of assessment methods if there is more than one group | *Page 5* | The evaluation items in this study included medical history (e.g., peripheral artery disease and diabetes mellitus), smoking history, age, etiology, defect size, defect site, venous congestion, necrosis, and flap infection. All clinical data were retrospectively obtained from medical records. Defect size was assessed based on operative findings. The presence of venous congestion in the flap was evaluated on the first postoperative day. |
| Bias | 9 | Describe any efforts to address potential sources of bias | Page 5 | This study was conducted using predefined clinical criteria for the indication of the DSNB technique, and all clinical data were collected from medical records using consistent definitions and evaluation methods. |
| Study size | 10 | Explain how the study size was arrived at | Page 5 | The study size was determined by the number of eligible patients treated during the study period. |

Continued on next page

| Quantitative variables | 11 | Explain how quantitative variables were handled in the analyses. If applicable, describe which groupings were chosen and why | Page 7 | All variables were analyzed as categorical variables, and no continuous variables were included in the statistical analysis. |
| --- | --- | --- | --- | --- |
| Statistical methods | 12 | (*a*) Describe all statistical methods, including those used to control for confounding | Page 7 | The relationship between complications, smoking history, and fractures with flap necrosis was examined. Because this involved categorical variables, the two-tailed Fisher’s exact test was used for the analysis. |
|  |  | (*b*) Describe any methods used to examine subgroups and interactions |  | Not applicable |
|  |  | (*c*) Explain how missing data were addressed | Page7.8 | No missing data were identified in the variables analyzed. |
|  |  | (*d*) *Cohort study*—If applicable, explain how loss to follow-up was addressed  *Case-control study*—If applicable, explain how matching of cases and controls was addressed  *Cross-sectional study*—If applicable, describe analytical methods taking account of sampling strategy | Page 7.8 | No loss to follow-up occurred during the study period, and no sensitivity analyses were performed. |
|  |  | (*e*) Describe any sensitivity analyses |  | Not applicable |
| Results | | | | |
| Participants | 13* | (a) Report numbers of individuals at each stage of study—eg numbers potentially eligible, examined for eligibility, confirmed eligible, included in the study, completing follow-up, and analysed | Page 8 | All 22 patients who underwent soft tissue defect reconstruction using the DSNB technique were followed up for at least 1 year. |
|  |  | (b) Give reasons for non-participation at each stage |  | Not applicable |
|  |  | (c) Consider use of a flow diagram |  | Not applicable |
| Descriptive data | 14* | (a) Give characteristics of study participants (eg demographic, clinical, social) and information on exposures and potential confounders | Page8.9 | Participant characteristics, including demographic (age, sex), clinical (medical history), and social factors (smoking history), as well as potential confounders (fibular fracture), are described in the Results section. |
|  |  | (b) Indicate number of participants with missing data for each variable of interest |  | Not applicable |
|  |  | (c) *Cohort study*—Summarise follow-up time (eg, average and total amount) | Page8 | All patients were followed up for at least 1 year. |
| Outcome data | 15* | *Cohort study*—Report numbers of outcome events or summary measures over time | Page8.9 | Outcome events, including flap necrosis, venous congestion, and infection, are reported in the Results section. |
|  |  | *Case-control study—*Report numbers in each exposure category, or summary measures of exposure |  |  |
|  |  | *Cross-sectional study—*Report numbers of outcome events or summary measures |  |  |
| Main results | 16 | (*a*) Give unadjusted estimates and, if applicable, confounder-adjusted estimates and their precision (eg, 95% confidence interval). Make clear which confounders were adjusted for and why they were included | Page8.9 | Unadjusted estimates of outcome events are presented in the Results section. No confounder-adjusted analyses were performed. |
|  |  | (*b*) Report category boundaries when continuous variables were categorized |  | Not applicable |
|  |  | (*c*) If relevant, consider translating estimates of relative risk into absolute risk for a meaningful time period |  | Not applicable |

Continued on next page

| Other analyses | 17 | Report other analyses done—eg analyses of subgroups and interactions, and sensitivity analyses | Page8.9 | No additional analyses were performed. |
| --- | --- | --- | --- | --- |
| Discussion | | | | |
| Key results | 18 | Summarise key results with reference to study objectives | Page9 | The flap survived in 22 patients, there were no cases of partial necrosis, and two patients (9.1%) had margin necrosis that did not require additional surgery. |
| Limitations | 19 | Discuss limitations of the study, taking into account sources of potential bias or imprecision. Discuss both direction and magnitude of any potential bias | Page12 | This study has several limitations. First, this was not a comparative study between the conventional RSAF and the DSNB technique, which limits the ability to draw definitive conclusions regarding their relative effectiveness. Second, the sample size was small, which may reduce the precision of the estimates. In addition, this study is subject to potential selection bias, as the indication for the DSNB technique was determined at the discretion of the surgeon, which may have influenced the outcomes. Furthermore, this study is limited by the presence of a learning curve associated with the surgical technique. The cases of venous congestion were observed in the early phase of the series, suggesting that the outcomes may have been influenced by technical inexperience and potentially underestimated. However, the magnitude of this effect is difficult to determine. |
| Interpretation | 20 | Give a cautious overall interpretation of results considering objectives, limitations, multiplicity of analyses, results from similar studies, and other relevant evidence | Page 13 | However, given the non-comparative design, small sample size, and potential sources of bias, these findings should be interpreted with caution within the scope of the present study objectives. The results are comparable to those reported for other modified RSAF techniques; however, as these analyses were exploratory, the present findings should be considered hypothesis-generating, and further studies are required to confirm these observations. |
| Generalisability | 21 | Discuss the generalisability (external validity) of the study results | Page12 | The generalisability of the present findings may be limited due to the single-centre design, small sample size, and institution-specific indications for the DSNB technique; therefore, caution is required when applying these results to other clinical settings or patient populations. |
| Other information | |  | | |
| Funding | 22 | Give the source of funding and the role of the funders for the present study and, if applicable, for the original study on which the present article is based | Page 14 | This research did not receive any specific grant from funding agencies in the public, commercial, or not-for-profit sectors. |

*Give information separately for cases and controls in case-control studies and, if applicable, for exposed and unexposed groups in cohort and cross-sectional studies.

**Note:** An Explanation and Elaboration article discusses each checklist item and gives methodological background and published examples of transparent reporting. The STROBE checklist is best used in conjunction with this article (freely available on the Web sites of PLoS Medicine at http://www.plosmedicine.org/, Annals of Internal Medicine at http://www.annals.org/, and Epidemiology at http://www.epidem.com/). Information on the STROBE Initiative is available at www.strobe-statement.org.
